# Effect of aromatic massage on brain waves and physiological indices of older adults

**DOI:** 10.1111/psyg.13153

**Published:** 2024-06-14

**Authors:** Mitue Sato, Yuu Koshu, Masahiro Sugimoto

**Affiliations:** ^1^ Department of Nursing Kiryu University Midori Japan; ^2^ Home Care Nursing Dokkyo Medical University Tochigi Japan; ^3^ Institute for Advanced Biosciences Keio University Yamagata Japan

**Keywords:** aroma, brain wave, immunoglobulin, massage, nursing research

## Abstract

**Background:**

Massage and aromatherapy are frequently used by older adults as alternative interventions to enhance immunity and induce relaxation. This pilot study evaluated the effect of massage therapy with oil and aromatherapy alone and in combination using objective biological indices.

**Methods:**

Twenty‐eight participants recruited by convenience sampling included adults aged between 25 and 65 years (Group 1), elderly individuals over 65 years without nursing care (Group 2), and older adults over 65 needing long‐term nursing support (Group 3). A multiple‐group pretest‐post‐test design was employed, and the effect among the three groups was compared. Interventions included: (i) oil massage therapy; (ii) aromatherapy; and (iii) aroma oil massage therapy. Each therapy session lasted 5 min, with 3 min of observation before and after the session and 10 min interval between sessions. Group 3 omitted one therapy (2: aromatherapy) to reduce their physical burden. An electroencephalogram (EEG) was recorded for α, β, and θ activities of brain waves. EEG data were collected at three points: before, during, and after each treatment. Salivary secretory immunoglobulin A (s‐IgA) concentration, oxygen saturation (SPO_2_), and pulse rate were measured before and after each session.

**Results:**

Across all therapy modalities, there was a noticeable increase in the α wave, indicative of relaxation, during the treatment. Significant differences were observed before and during the oil massage in both Group 1 and Group 2. Aromatherapy demonstrated a significant difference before and during treatment in Group 1. Among the biological parameters, s‐IgA levels indicated no significant changes. The pulse rate decreased with oil massage. Significant differences were noted before and after therapy in all cases for SPO_2_ and in Group 2 for pulse rate.

**Conclusions:**

Three therapies induced EEG and physiological changes in the adult group and older adults without nursing care. However, these effects are limited in older adults requiring nursing care.

## INTRODUCTION

Symptoms and indicators such as incontinence, delirium, and dysphagia tend to escalate with advancing age. However, the risk of autonomy loss for older adults does not stem directly from these diseases. Although these syndromes are prevalent among older adults, their intricate nature poses challenges for effective treatment using modern medicine. Therefore, various complementary and alternative therapeutic approaches have been proposed. Non‐pharmacological interventions have been implemented, including animal,^1^ active reminiscence,^2^ and humour therapies.^3^ Substantive objective evidence has been provided for each therapeutic modality.

As an alternative intervention, massage therapy has demonstrated the potential to enhance the health and well‐being of older adults and possibly foster increased family engagement in caregiving.^4^ However, a comprehensive review of nine studies investigating massage therapy in older adults with dementia revealed significant reductions in the Cohen‐Mansfield Agitation Inventory (CMAI), addressing agitation, anxiety, and aggressive behaviour. However, no significant alterations were observed in the Neuropsychiatric Inventory or Mini‐Mental State Examination to assess cognitive function.^5^ Consequently, despite massage therapy's many benefits, variations in techniques, duration, and measured outcomes exhibit inconsistent effects.

Substantial evidence supports aromatherapy as a viable alternative technique which affects physical, mental, and psychological facets across diverse populations. Aromatherapy effectively reduces sympathetic activity, augments parasympathetic activity, alleviates stress, facilitates muscle relaxation, and enhances sleep quality. Additionally, it mitigates anxiety, depression, and insomnia.^6^ Notably, blended lavender and lemon balm oil effectively reduced dyspnoea perception in older adults.^7^ Olfactory stimulation through aromatherapy has shown promise in ameliorating cognitive symptoms in patients.^8^ Furthermore, aromatherapy shows benefits for older patients with chronic pain. It reduces pain scores and emotional distress.^9^ Extensive research has explored the efficacy of aromatherapy in enhancing sleep quality and reducing stress.^10^ Aromatherapy has been shown to positively affect various cognitive functions in older persons with dementia.^11^ Aromatherapy‐induced fatigue reduction has been evaluated using scales such as the State–Trait Anxiety Inventory and Visual Analogue and Face Scales.^9^

Polytherapy combines multiple therapeutic methods to achieve greater efficacy.^12^ Simultaneous aromatherapy and massage improved scores in the Hospital Anxiety and Depression Scale, which assesses anxiety and depression symptoms.^13^ These data indicate that massage therapy has been assessed to be better than rest regardless of the essential oils used. These results are based on subjective/psychological not physiological/objective assessment. Furthermore, aromatherapy body treatments provide a more substantial and sustained reduction in fatigue, especially mental fatigue, than massage alone.^9^ Foot massage with lavender essential oil for older adults improves sleep quality.^14^ Studies have shown that combining multiple therapies is more effective than individual ones.

Most evidence for alternative therapies is based on studies on changes in the measures of the expected effects of each. However, in recent years, increasing efforts have been made to monitor the physiological changes more directly to provide evidence for each therapy. One study that evaluated the stress‐reducing effects of aromatherapy in older adults requiring care examined changes in blood pressure and cyclic changes using a portable device. It found that patients could remain relaxed for 30% of their active time.^15^ In a meta‐analysis of studies on short‐term improvements in mood and immunity using salivary immunoglobulin A (s‐IgA) concentration, interleukin‐6 production, and natural killer cell activation as assessment methods, the authors reported that trends toward improvement were observed.^16^ However, these were not statistically significant due to small sample sizes.^17^ Regarding aroma head therapy's effects, no differences were found in mental stress indices before and after massage. However, electroencephalogram (EEG) amplitude is significantly decreased in the right brain.^17^ Salivary s‐IgA was measured in older adults with dementia, along with mental status and activity scale measures related to progressive muscle relaxation, which improved behavioural and psychological symptoms of dementia and activities of daily living but had no effect on immune function.^18^

This pilot study evaluated the effects of massage therapy with oil and aromatherapy alone and in combination using objective biological indices. The participants included adults aged between 25 and 65 years (Group 1), older adults over 65 years without nursing care (no certification of needing long‐term care institutionalisation in Japan, Group 2), and older adults over 65 years requiring long‐term nursing support (certification of needing long‐term care Level 3 and higher institutionalisation in Japan, Group 3). Changes in relaxation and arousal levels were evaluated by EEG observation, including α, β, and θ activities of the brain. Salivary s‐IgA, oxygen saturation (SPO_2_), and pulse rate were measured to evaluate therapy effects among the three groups.

## METHODS

### Participants

This study was conducted following the Declaration of Helsinki. The Ethics Review Committee of Tohto University approved the study (Approval no. F20087, March 26, 2021). Written informed consent was obtained from all participants. For Group 3, the research plan was explained to the participants and their families.

Group 1: Adults aged between 25 and 65 years (*n* = 11).

Group 2: Adults aged 65 and older. The participants lived at home (no certification of needing long‐term care) (*n* = 9).

Group 3: Older adults aged 65 and older who require nursing care provided by long‐term care insurance (certification of needing long‐term care Level 3 and higher). The participants lived in special older adult care facilities (*n* = 8).

In Japan, nursing care is certified at age 65. Nursing care is supported for older adults who need it. Group 1 was eligible for long‐term care certification. Group 2 included patients who could live independently. Group 3 included those requiring long‐term care (Level 3) under nursing care for older adults. The conditions for Level 3 of this certification were: (i) physical function limitations in daily living activities (eating, bathing, using the toilet, dressing, and moving); (ii) cognitive limitations (memory, judgement, cognitive ability, etc.); and (iii) environmental limitations (difficulty living alone safely). Convenience sampling was used for recruitment.

### Intervention

A multiple‐group pretest‐post‐test design was employed. The study involved the implementation of three therapeutic interventions:

1. oil massage therapy
2. aromatherapy
3. aroma oil massage therapy.

**Figure 1:**
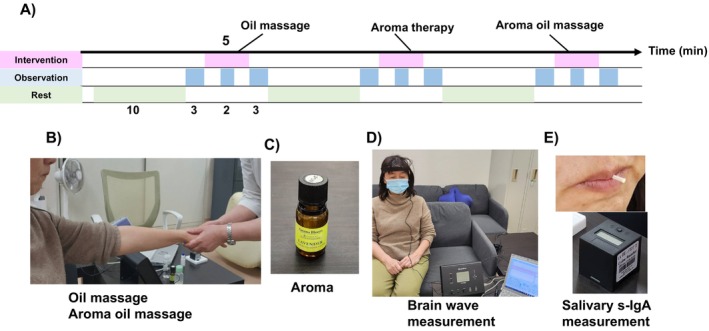
Intervention study protocol. (a) Relationship between each treatment and interval. Oil massage, aromatherapy, and aroma oil massage were used as interventions for 5 min each. Observations were conducted for 3 min before and after each session, with 10‐min intervals between each. Electroencephalogram (EEG) data were collected at three points: at the beginning of each treatment, 2 min after the beginning, and at the end of each treatment. Biological samples were collected at two points: at the beginning and at the end of each treatment. Note that aromatisation and one interval were omitted in Group 2. (b) The performance of oil massage and aroma + oil massage. The massage was performed on the whole forearm. (c) Container with aroma. (d) EEG measurement. (e) Saliva collection and salivary immunoglobulin A (s‐IgA) measurement device.

Each intervention session lasted 5 min. Before and after each session, 3 min were used for observation. The rest between each interval was 10 min (Fig. 1A). These therapies were performed in quiet rooms. The aromatherapy was conducted in a different room to minimise the lingering scent. Therefore, participants were required to change rooms after Session 1 and return to the first room after Session 2. The second intervention (aromatherapy) was omitted for Group 3 to reduce the experiment's duration. They received only the first and third interventions.

The forearms were massaged in interventions 1 and 3. If the right arm was massaged in intervention 1, the left arm was massaged in intervention 3, or vice versa (Fig. 1B). A single therapist conducted all massage sessions to ensure consistency in massage technique and intensity. During the massage, one hand secured the participants's wrist while the other extended the elbow joint, released tension in the upper body, and simultaneously elevated it by rubbing.

The essential oil for aromatic therapy was undiluted *Lavandula angustifolia* (genuine lavender) (Planarom, Yamanashi, Japan). Undiluted oils were dropped into an unglazed pot. The participants held the pot with one hand, slowly shaking it approximately 15 cm from their faces while inhaling its fragrance. Aromatic massage was conducted with a 0.3% solution of jojoba (MUJI, Tokyo, Japan) as the carrier oil (Fig. 1C). These interventions were conducted between 10:00 and 14:00 hours, maintaining a room temperature of 25–26°C and humidity at approximately 50%. Each participant was administered the test individually in a quiet room.

### Observations

Electroencephalography (EEG) and physiological indices were recorded for each treatment group. EEG observations were made before and after each treatment and 2 min after the start of each treatment. The participants were required to close their eyes during the EEG measurements to prevent environmental disturbances.

#### EEG

The EEG was measured using Brain Pro FM‐929 (FUTEK Electronics Co., Ltd., Kanagawa, Japan), with a sensor pad attached to the forehead (Fig. 1D). The electrodes were placed in the frontal area (Fp1) according to the International Electrode Placement Method (10–20 method), and a unipolar induction method was used with an unrelated electrode at the earlobe. Four to 7.5 Hz was measured as θ, 8 to 13 Hz as α, and 13 to 30 Hz as β waves. The content‐accumulated values were obtained.

#### Physiological indices

Non‐stimulated whole saliva was collected, and s‐IgA was quantified using a cube reader (SOMA Biosciences, Oxford, UK) (Mitsuishi, Okamura, *et al*. 2023).^19^ The saliva collection kit (oral fluid collection) was held in the mouth for approximately 1 min until the collection swab was soaked with saliva. After collection, the saliva was immediately placed in a bottle and slowly mixed without foaming. The s‐IgA concentration was determined using a cube reader (Fig. 1E). Oxygen saturation (SPO_2_) and resting pulse rate were measured using an oximeter (Pulse Oximeter OX‐200; Dretec Co., LTD, Saitama, Japan).

### Data analysis

A heatmap was used to visualise changes in the quantitative data. Wilcoxon matched‐pairs signed rank test evaluated the observed data before and after each intervention. Data before and during each intervention were also evaluated. GraphPad Prism software (ver. 9.5.1; GraphPad Software Inc., San Diego, CA, USA) and MeV TM4 (v. 4.9.1) were used. Individual data are visualised using line‐dot plots. Statistical significance was set at *P* < 0.05.

## RESULTS

The means and SD for age were 38.2 ± 14.0 for Group 1, 77.7 ± 6.00 for Group 2, and 82.6 ± 7.25 for Group 3. The ratios of males/females per group were 1:10, 6:3, and 1:7, respectively.

**Figure 2:**
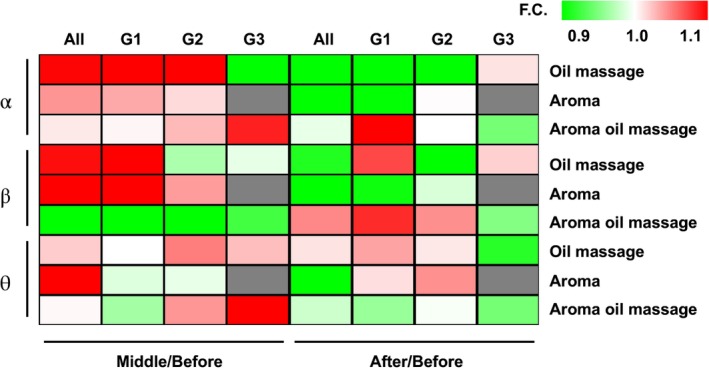
Heatmap showing changes in electroencephalogram (EEG) with each therapy. The left half shows the change during/before therapy. The right half shows the changes after/before therapy. From top to bottom, α, β, and θ are shown. All indicates all cases; G1, G2, and G3 indicate Group 1, Group 2, and Group 3, respectively. The colour bar in the upper right corner indicates the colour according to the ratio of change in each value (fold change, F.C.): red for values greater than 1, yellowish‐green for values less than 1, and white for values equal to 1.

Figure 2 illustrates the EEG changes in the heatmap. The left half of the figure shows the ratio during and before therapy; the right half shows the ratio after and before therapy. Red and green indicate upward and downward trends in the observed data, respectively. The wave data are broadly red during all therapies, indicating an upward trend. Oil massage is associated with an upward trend in all cases (Groups 1 and 2) and a decreasing trend in Group 3. In aromatherapy, α waves showed increasing trends in all cases in Group 1 and Group 2. The β‐wave changes were higher in all cases of oil massage and aromatherapy and Group 1, while the θ waves were higher in Group 3 for aromatherapy and oil massage. The right half of the figure shows the after/before therapy ratios. The α‐wave change was high in Group 1 for aroma oil massage. The β‐wave change showed high trends in Group 1 of oil and aroma oil massages. The θ‐wave change showed high trends in Groups 1 and 2 of oil massage and aromatherapy.

**Figure 3:**
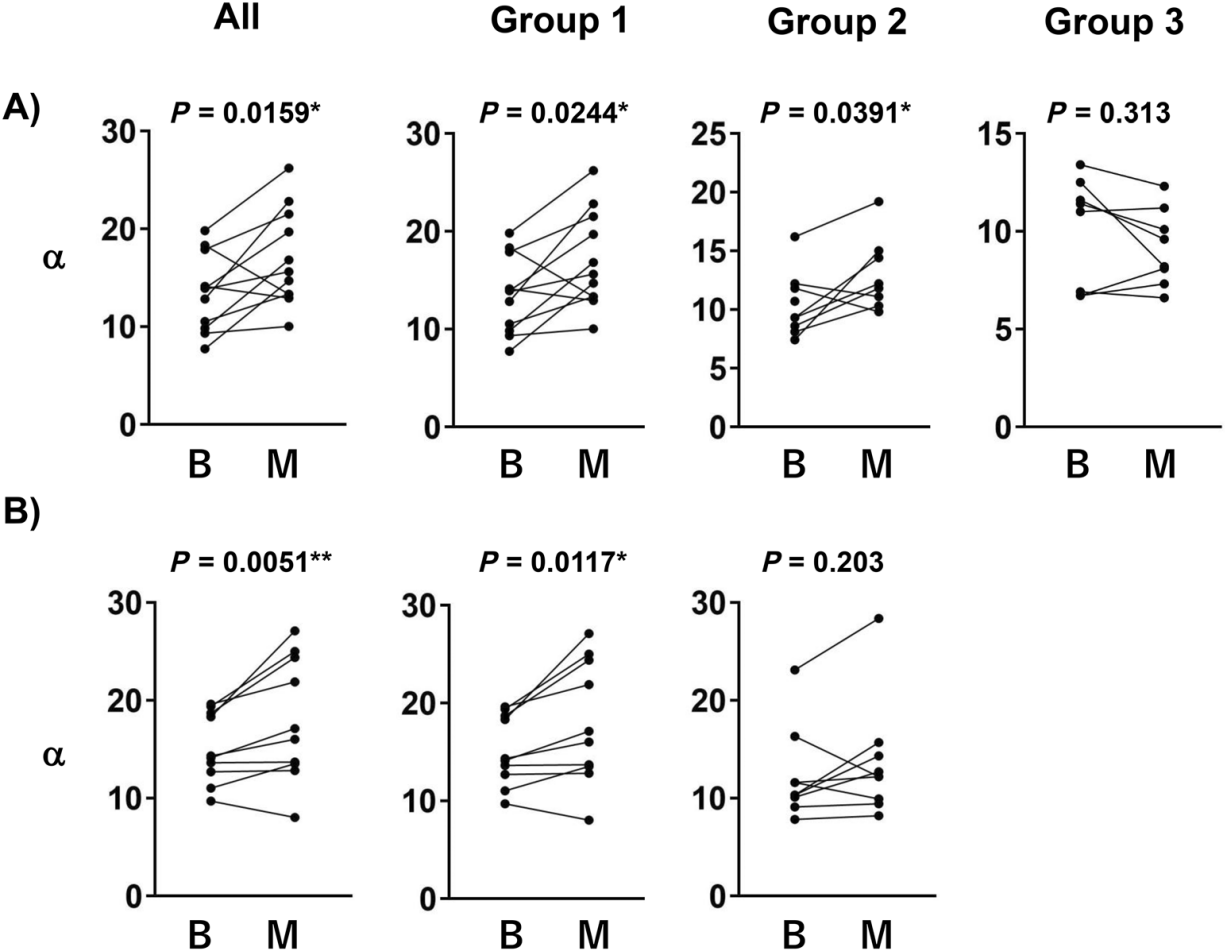
Line‐dot plots showing the change in α waves during/before therapy in oil massage and aromatherapy. (a) Oil massage, (b) aromatherapy. B and M indicate before and middle, respectively. All values have no unit. *P < 0.05, **P < 0.01 (Wilcoxon matched‐pairs signed rank test).

Figure 3 illustrates the variables that differed significantly according to intervention. The changes in α wave before and during oil massage are depicted in Fig. 3A. A significant change (*P* = 0.0159) was observed in all cases, with an increasing trend, except for two cases. Significant changes were also observed (*P* = 0.244 for Group 1 and *P* = 0.0391 for Group 2), indicating an increasing trend. However, several cases showed opposite ones. Group 3 showed no significant change (*P*= 0.0313). Figure 3B illustrates the changes in the α wave before and during the aromatherapy. The α wave significantly increased with *P* = 0.0051 for all patients and *P* = 0.0117 for Group 1. Patients with the lowest preoperative values showed lower intraoperative ones. Group 2 showed no significant change (*P* = 0.203).

**Figure 4:**
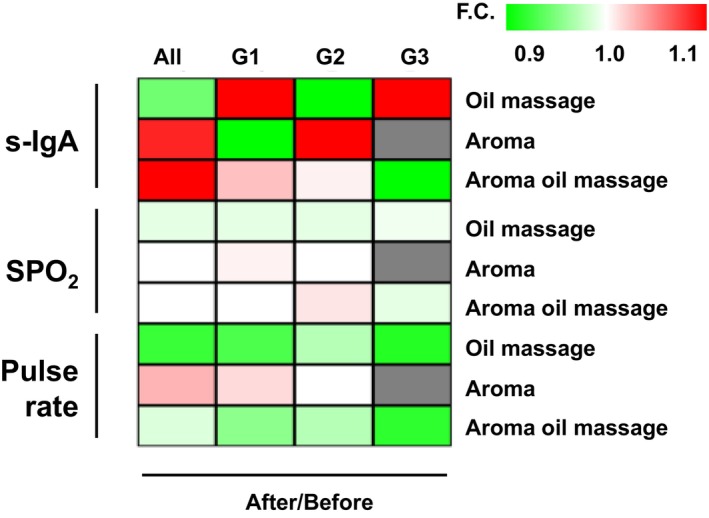
Heatmap showing the change in each biometric index with each treatment. After data/before data‐changes (fold change, F.C.) are shown. From top to bottom: salivary immunoglobulin A (s‐IgA), oxygen saturation (SPO_2_), and pulse rate. All indicates all cases; G1, G2, and G3 indicate Group 1, Group 2, and Group 3, respectively. The colour bar in the upper right corner indicates the colour according to the ratio of change of each value: red for values above 1, yellowish‐green for values below 1, and white for values of 1.

Figure 4 illustrates the physiological index changes. From top to bottom, the figure displays the observation ratio before and after treatment, including s‐IgA, SPO_2_, and pulse rate. Notably, s‐IgA changed substantially, while SPO_2_ and pulse rate changes were relatively minor.

**Figure 5:**
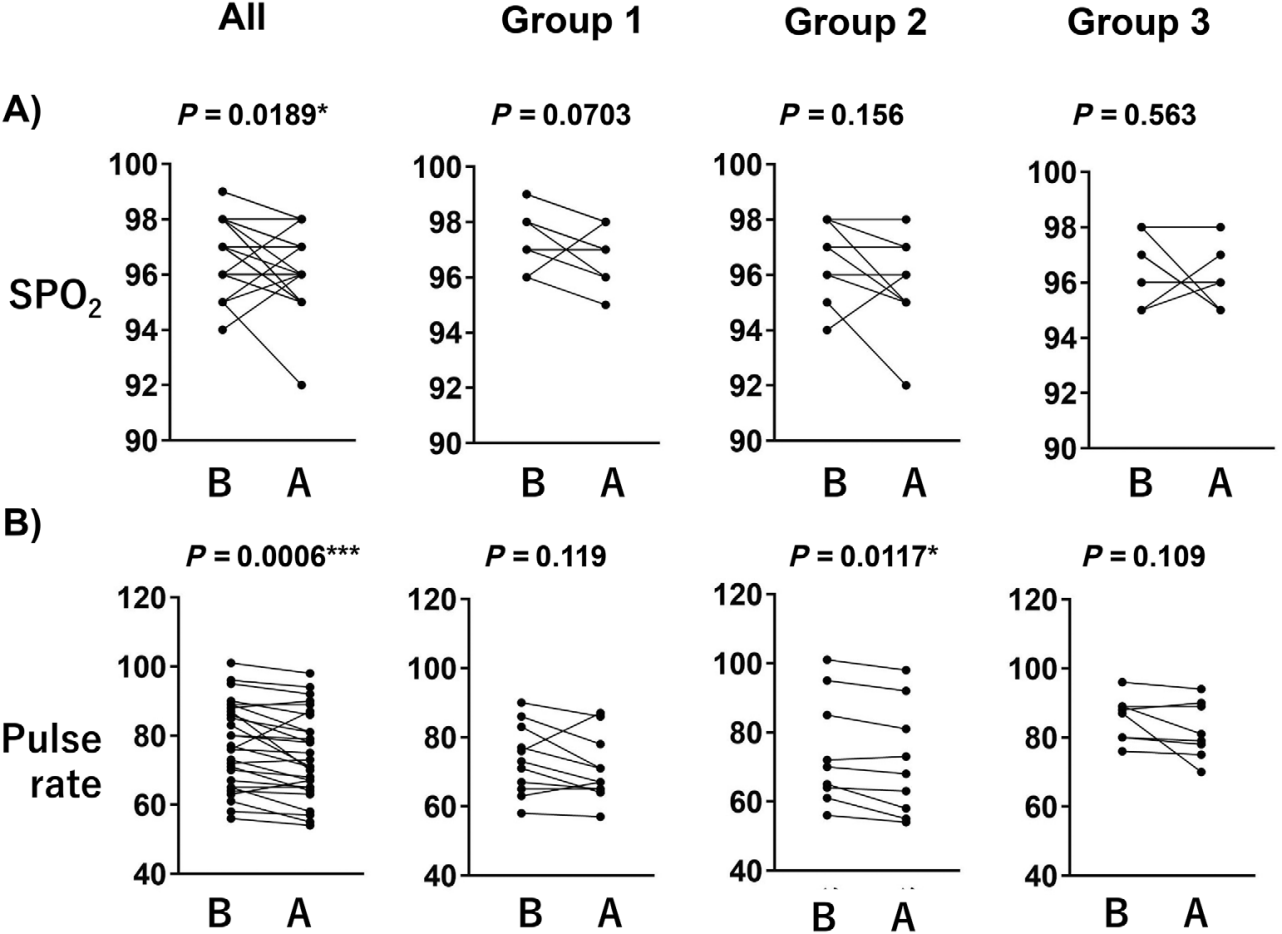
Line‐dot plots showing the change of oxygen saturation (SPO_2_) (%) and pulse rate (beats per minute) in oil massage after/before therapy. (a) Oil massage, (b) aromatisation. B and A indicate before and after, respectively. *P < 0.05, **P < 0.01 (Wilcoxon matched‐pairs signed rank test).

Figure 5 illustrates the variables that exhibited significant differences. Figure 5A depicts the change in SPO_2_ during oil massage therapy. The overall trend indicated a downward trend with a significant difference of *P* = 0.0189 for all cases. Each group demonstrates a decreasing trend, with Group 1 exhibiting a smaller value of *P* = 0.0703 compared to the other groups despite no significant level. Figure 5B depicts the change in pulse rate during oil massage therapy. A decreasing trend was observed, with a significant difference of *P* = 0.0006 for all cases. However, Groups 1 and 3 did not exhibit any significant changes.

## DISCUSSION

Oil massage, aromatherapy, and aroma oil massage were administered to adults and older adults to assess their effects on EEG and physiological indices. EEG data were recorded before, during, and after each therapy session, and other biological indices were measured before and after each session. Wave activity tended to increase during aroma oil massage during treatment compared to pretreatment. However, the older adult group requiring nursing care displayed a decreasing wave trend. The α waves increased during relaxation, β increased during arousal and external stimulation, and θ increased during relaxation and the vagal state, suggesting that the oil massage relaxed the brain.

While alternative therapies ideally should be effective across different cases, individual variations in responsiveness may exist in practice. For example, approximately 10% of participants reported discomfort with massage therapy.^20^ In this study, older adults needing nursing care responded differently from the other groups, with weaker effects observed for each therapy.

In this study, each therapy session was set to last 5 min. This duration facilitated evaluating the effects of shorter therapy. However, other studies on similar alternative therapies assessed outcomes over prolonged periods. For instance, a 4‐week study on aromatherapy demonstrated a reduction in pain levels and recovery from unpleasant emotions in older adults with chronic pain.^21^ Muscle relaxation for 90 days in older adults with dementia resulted in behavioural changes without a concurrent impact on immunity.^18^ A 2‐week intervention involving aromatherapy combined with massage improved constipation in older patients.^22^ In contrast, our study focused on changes in EEG and physiological indices for a short‐term intervention. This approach minimised the effects of various external factors that may occur during a longer study period. The variables employed in our observations are likely to offer insights into short‐term therapy's effectiveness.

The decreased oxygen saturation and pulse rate observed in the oil massage group in ageing individuals who did not require nursing care indicated a relaxing effect. Conversely, no changes in s‐IgA levels were evident in any group or treatment. While long‐term changes in s‐IgA indicate immunity, they may also reveal short‐term stress levels. For example, s‐IgA concentrations increase during pleasant mood states.^23^ The concentration of s‐IgA and salivary secretion increased after traditional Chinese exercise.^24^ Aromatherapy administered to healthy individuals over 6 weeks activates the immune and autonomic nervous systems, resulting in an increased concentration of s‐IgA.^25^ However, no significant changes in s‐IgA levels were observed in this study. Furthermore, massage for cancer survivors, while reducing subjective physical distress, did not significantly change s‐IgA levels.^26^ These findings contrast with those of the present study, in which no significant changes in s‐IgA levels were identified.

This study has several limitations. This is a pilot study, and the sample size for each group was small. The theoretical sample size was not estimated since no treatment was conducted before this study. Further validation using a larger sample size is required. Group 3 omitted the aromatherapy and one interval. Before the aromatherapy treatment, the subjects are required to move to another room and move back to the same room after the treatment. Consistent treatment should be offered to all three groups to more rigorously evaluate the effect of aromatherapy. EEG and bioindicators exhibited the most significant changes following the initial oil massage, surpassing the effects observed with subsequent therapies. This result raises concerns regarding potential dependencies during each therapy session. Exploring the effect of varying the treatment sequence to mitigate potential order‐related biases is essential. As investigated here, short‐term therapies should not have a pronounced effect on immunity. This study emphasised changes in relaxation and stress. This study also highlights the need to evaluate the effects of long‐term therapies.

## CONCLUSIONS

This pilot study aims to evaluate the effects of massage, aromatherapy, and aroma oil massage on older adults. EEG and physiological index changes revealed a tendency for α, β, and θ waves to increase during therapy sessions. Significant changes were observed in Groups 1 and 2 during oil massage and in Group 1 during aromatherapy. Notably, no significant changes were observed in s‐IgA among the physiological indicators. However, SPO_2_ and pulse rate decreased during oil massage. Similar EEG and physiological indicator changes were anticipated in older adults requiring nursing care. However, validation through long‐term therapy is imperative for a comprehensive understanding.

## FUNDING INFORMATION

This work is supported by the KAKENHI (JP19K11202).

## DISCLOSURE

The authors have no potential conflicts of interest to disclose.

## ETHICS APPROVAL STATEMENT

The Ethics Review Committee of Tohto University approved this study (Approval no. F20087, March 26, 2021).

## PATIENT CONSENT STATEMENT

Written informed consent was obtained from all the participants. For elderly individuals over 65 requiring nursing care, the research plan was explained to the participants and their families.

## Data Availability

The data that support the findings of this study are available from the corresponding author upon reasonable request.

## References

[1] Kawamura N , Niiyama M , Niiyama H . Long‐term evaluation of animal‐assisted therapy for institutionalized elderly people: a preliminary result. Psychogeriatrics 2007; 7: 8–13.

[2] Yamagami T , Oosawa M , Ito S , Yamaguchi H . Effect of activity reminiscence therapy as brain‐activating rehabilitation for elderly people with and without dementia. Psychogeriatrics 2007; 7: 69–75.

[3] Takeda M , Hashimoto R , Kudo T *et al*. Laughter and humor as complementary and alternative medicines for dementia patients. BMC Complement Altern Med 2010; 10: 1–7. 20565815 10.1186/1472-6882-10-28 PMC2896339

[4] McFeeters S , Pront L , Cuthbertson L , King L . Massage, a complementary therapy effectively promoting the health and well‐being of older people in residential care settings: a review of the literature. Int J Older People Nurs 2016; 11: 266–283. 26875503 10.1111/opn.12115

[5] Margenfeld F , Klocke C , Joos S . Manual massage for persons living with dementia: a systematic review and meta‐analysis. Int J Nurs Stud 2019; 96: 132–142. 30679034 10.1016/j.ijnurstu.2018.12.012

[6] Passos NN , Campanelli SE , da Silva CR , da Silva França RC , de Sousa Rosso ICA . Psychological and neurophysiological effects of inhaled aromatherapy. Res Soc Devel 2022; 11: e442111436361‐e.

[7] Ebihara T , Yamasaki M , Kozaki K , Ebihara S . Medical aromatherapy in geriatric syndrome. Geriatr Gerontol Int 2021; 21: 377–385. 33789361 10.1111/ggi.14157

[8] Cha H , Kim S , Seo M‐s , Kim H‐s . Effects of olfactory stimulation on cognitive function and behavior problems in older adults with dementia: a systematic literature review. Geriatr Nurs 2021; 42: 1210–1217. 34425423 10.1016/j.gerinurse.2021.07.003

[9] Takeda H , Tsujita J , Kaya M , Takemura M , Oku Y . Differences between the physiologic and psychologic effects of aromatherapy body treatment. J Altern Complement Med 2008; 14: 655–661. 18637761 10.1089/acm.2007.0591

[10] Her J , Cho MK . Effect of aromatherapy on sleep quality of adults and elderly people: a systematic literature review and meta‐analysis. Complement Ther Med 2021; 60: 102739. 34166869 10.1016/j.ctim.2021.102739

[11] Jimbo D , Kimura Y , Taniguchi M , Inoue M , Urakami K . Effect of aromatherapy on patients with Alzheimer's disease. Psychogeriatrics 2009; 9: 173–179. 20377818 10.1111/j.1479-8301.2009.00299.x

[12] Ke M‐H , Hsieh K‐T , Hsieh W‐Y . Effects of aromatherapy on the physical and mental health and pressure of the middle‐aged and elderly in the community. Appl Sci 2022; 12: 4823.

[13] Mehrabian S , Tirgari B , Forouzi MA , Tajadini H , Jahani Y . Effect of aromatherapy massage on depression and anxiety of elderly adults: a randomized controlled trial. Int J Ther Massage Bodywork 2022; 15: 37–45. 35280245 10.3822/ijtmb.v15i1.645 PMC8887855

[14] Puspawati NLPD , Santi NKA , Saraswati NLGI . The effect of foot massage with lavender essential oil on the sleep quality of the elderly in banjar gelumpang, sukawati village. Basic Appl Nurs Res J 2021; 2: 47–51.

[15] Kikukawa H , Koura S , Kuge M , Miwa K , Yao M . Effects of aromatherapy of Himekuromoji (Lindera lancea), essential oil on vital and stress, emotion and cognitive function of the elderly in need of care. Open J Ther Rehab 2021; 9: 83–97.

[16] Ayling K , Sunger K , Vedhara K . Effects of brief mood‐improving interventions on immunity: a systematic review and meta‐analysis. Psychosom Med 2020; 82: 10–28. 31609922 10.1097/PSY.0000000000000760

[17] Jeong J , Lee S , Jang J , Bang H . Effects of aroma head therapy on stress and brain wave change using EEG. Revista De Psicología Del Deporte (J Sport Psychol) 2021; 30: 133–140.

[18] Ikemata S , Momose Y . Effects of a progressive muscle relaxation intervention on dementia symptoms, activities of daily living, and immune function in group home residents with dementia in Japan. Jpn J Nurs Sci 2017; 14: 135–145. 27696678 10.1111/jjns.12147 PMC5396310

[19] Mitsuishi H, Okamura H, Moriguchi Y, Aoki Y . The validity of the salivary cortisol analysis method using the Cube reader in Japanese university students. Jpn Psychol Res 2023; 65: 369–378.

[20] Cambron JA , Dexheimer J , Coe P , Swenson R . Side‐effects of massage therapy: a cross‐sectional study of 100 clients. J Altern Complement Med 2007; 13: 793–796. 17983334 10.1089/acm.2006.6401

[21] Tang SK , Tse M . Aromatherapy: does it help to relieve pain, depression, anxiety, and stress in community‐dwelling older persons? Biomed Res Int 2014; 2014: 1–12. 10.1155/2014/430195 PMC4119713 25114901

[22] Lafcı D , Kaşikçi M . The effect of aroma massage on constipation in elderly individuals. Exp Gerontol 2023; 171: 112023. 36372282 10.1016/j.exger.2022.112023

[23] Stone AA , Cox DS , Valdimarsdottir H , Jandorf L , Neale JM . Evidence that secretory IgA antibody is associated with daily mood. J Pers Soc Psychol 1987; 52: 988–993. 3585705 10.1037//0022-3514.52.5.988

[24] Bayat‐Movahed S , Shayesteh Y , Mehrizi H *et al*. Effects of qigong exercises on 3 different parameters of human saliva. Chin J Integr Med 2008; 14: 262–266. 19082797 10.1007/s11655-008-0262-6

[25] Takagi C , Nakagawa S , Hirata N , Ohta S , Shimoeda S . Evaluating the effect of aromatherapy on a stress marker in healthy subjects. J Pharm Health Sci 2019; 5: 1–7. 10.1186/s40780-019-0148-0 PMC6693249 31428439

[26] Donoyama N , Satoh T , Hamano T , Ohkoshi N , Onuki M . Physical effects of Anma therapy (Japanese massage) for gynecologic cancer survivors: a randomized controlled trial. Gynecol Oncol 2016; 142: 531–538. 27430394 10.1016/j.ygyno.2016.06.022

